# Sense of Purpose Following a Dementia Diagnostic Appointment: Comparing Self- and Other-Reports of Care Recipients and Care Partners

**DOI:** 10.3389/fpsyg.2021.703478

**Published:** 2021-08-13

**Authors:** Matthew J. Wynn, Catherine H. Ju, Patrick L. Hill

**Affiliations:** Department of Psychological and Brain Sciences, Washington University in St. Louis, St. Louis, MO, United States

**Keywords:** purpose, dementia, Alzheimer, care partner, caregiving, observer-report

## Abstract

**Objective**: Purpose in life tends to decline in older adulthood and it is thought that intact cognitive functioning is required for purposeful living. Thus, it is likely that individuals may perceive older adults who are experiencing cognitive declines associated with dementia as having a reduced sense of purpose. Biases such as these may influence how individuals, especially care partners, interact with those with dementia.

**Method**: This study examined how sense of purpose changed following a dementia diagnostic appointment for both the person receiving a diagnosis and their care partner. This study also explored how each individual perceived the other member of the dyad’s sense of purpose. Older adults (47 care recipients and 75 care partners, 57% female; *M_age_* = 68.5 years, *SD_age_* = 12.0 years) provided self- and other-report ratings of sense of purpose before and after their appointment at a specialized memory clinic.

**Results**: Overall, both care recipients and care partners’ sense of purpose declined following a dementia diagnostic appointment [*t*(85) = 7.01, *p* < 0.001]. However, when comparing self-reports and other-reports of purpose, care partners reported that care recipients experienced a lower sense of purpose in life than the care recipients reported about themselves.

**Conclusions**: Care recipients and partners reported less purpose in life following their dementia diagnostic appointment. Care partners may hold certain biases regarding sense of purpose toward care recipients. These findings can inform future work regarding how care recipients and care partners can plan purposeful lives following a dementia diagnosis.

## Introduction

Previous research suggests that adults tend to believe that, when considering lifespan trajectories, people will tend to stop increasing on purposefulness in older adulthood, and potentially start to decline (Heckhausen et al., 1989). This perception appears to align with existing studies on sense of purpose during older adulthood. Indeed, meta-analytic work suggests a negative age association with purpose (Pinquart, 2002), which matches cross-sectional studies finding that younger and middle adults tend to report higher sense of purpose than older adults (Ryff, 1989; Ryff and Keyes, 1995). Moreover, longitudinal work finds that older adults tend to decline over repeated assessments on purpose (Hill and Weston, 2019; Lewis and Hill, 2020).

This perception also likely reflects the commonly held perception that older adulthood is a period of widespread declines (Sneed and Whitbourne, 2005). Purposefulness may be particularly contrary to older adult perceptions, given that having a sense of purpose involves greater engagement with life toward one’s broader life direction (Ryff, 1989; Scheier et al., 2006). Although life engagement is seen as a cornerstone of successful aging (Rowe and Kahn, 1997), it may be complicated by age-graded physical limitations and losses. Indeed, self-rated health was one of the few predictors of trajectories of change for sense of purpose in past work (Hill and Weston, 2019).

Given the known age-graded cognitive declines (Salthouse, 2009, 2012), it is also important to note that having a purpose in life is thought to require intact cognitive functioning (McKnight and Kashdan, 2009). Purposeful living likely requires frequent practice of cognitive functions in order to plan toward the future and organize life activities around pursuit of one’s goals. Researchers have noted that having a sense of purpose promotes a wide array of positive aging outcomes (Pfund and Lewis, 2020); for instance, adults who report a greater sense of purpose tend to outperform peers on measures of memory and executive functioning (Windsor et al., 2015; Lewis et al., 2017), and sense of purpose has been shown to longitudinally predict reduced risk for non-normative cognitive decline (Boyle et al., 2010). As such, it may be the case that people will be even more likely to expect reduced purposefulness for older adults who also are experiencing cognitive difficulties. Such biases may be particularly problematic when held by individuals responsible for decisions regarding the older adult’s care and daily life, and efforts to maintain purposeful engagement may be hindered when care partners make decisions based on a biased opinion that the older adult has little direction for life.

Research on this front, though, is relatively limited, as is work more broadly on whether people can reliably report on another person’s sense of purpose. It may be difficult for individuals to know whether another person perceives a direction for life, unless discussions about life goals are held regularly. Perhaps for this reason, the limited work on this topic has focused on married couples reporting on each other’s sense of purpose (Schmutte and Ryff, 1997). That research demonstrates relatively strong positive correlations between the individual’s report for sense of purpose and their partner’s perception. However, these associations were far from unity and thus suggest that discordance may occur even among close individuals. To understand this discordance requires getting both members of a dyad to provide self- and other-reports of purpose, allowing insights into whether discordance is higher or lower based on dyadic member status (here, care partner or care recipient).

This point is particularly important to consider in the context of older adults showing symptoms of cognitive decline and incipient dementia. This group is vulnerable both to losing primary decision-making for their lives, and to a growing concern that their care partners may not understand their needs and goals. Accordingly, it is valuable to consider whether care partners hold biased or inaccurate views of their recipients’ sense of purpose, particularly at the start of potential treatment or decision-making about the recipients’ cognitive issues. Based on past work with married couples (Schmutte and Ryff, 1997), one would expect reasonable accuracy in the care partners’ reports, insofar that they should be moderate-to-strongly correlated with self-reports from the older adult experiencing declines. However, given the beliefs that older adults decline in purposefulness with age (Heckhausen et al., 1989) and the assumption that purposeful living involves intact cognitive functioning (McKnight and Kashdan, 2009), mean-level biases are likely insofar that the care partner may report lower sense of purpose relative to the self-report.

### Current Study

The current study investigated that these claims with a sample of individuals attending an initial cognitive decline clinic visit as a result of personal or observer-reported cognitive concerns. Hereafter, we refer to these dyads as the care recipient and care partner. It should be noted that the required attendee may not be the ultimate primary care partner, and the visit may suggest little need for intervention or care. However, this attendee was the primary informant for the process of diagnosing cognitive issues, and if such a diagnosis were rendered, would be the individual with whom the future care process was discussed. As such, we employ the partner and recipient terminology both for parsimony, and because this framing reflects the most likely future roles based on the information available prior to the clinic visit.

Both members of the dyad provided self- and observer-reports for sense of purpose at an initial assessment before the appointment and again as close as possible following the appointment. Tests of the primary hypotheses – whether accuracy and biases in reporting were evidenced – concern primarily the initial assessment. However, we also discuss the reports over both assessments to explore the stability for sense of purpose following a potential major life event, namely the initial visit to the clinic and potential diagnosis of dementia.

## Materials and Methods

### Participants

Participants presented for an initial diagnostic assessment at a specialized dementia assessment clinic. About a month before the appointment date, a research assistant called the care partner to obtain consent and to give verbal consent for the care recipient to be contacted. The care recipient then received a call in which they gave their consent to participate. After obtaining consent from participants, a comprehensive copy of the consent form was emailed or mailed to the care partner and recipient. Due to concerns regarding potential participants’ cognitive capacities, care partners were the primary source of contact. If the care partner indicated the person would be unable to consent, proxy consent was obtained from the care partner along with assent from the recipient before continuing with study procedures.

Seventy-five care partners and 47 care recipients agreed to participate in the pre-appointment interview. Fifty-three (70.7% of the original sample) care partners and 33 (71.7% of the original sample) care recipients completed the post-appointment interview (Table 1). Participants who could not answer questions due to cognitive limitations (*n* = 7) were excluded from analysis. According to care recipient and care partner reports, 12 care recipients were suspected of having dementia or Alzheimer disease (36.4%), nine care recipients were suspected of having problems with memory and thinking (27.3%), and 12 care recipients reported being told they did not have any issues with dementia, Alzheimer disease, or any general memory problems (36.4%).

**Table 1:** Sample characteristics.

	*M*/*n*	*SD*/%
Age	68.53	12.03
**Gender**		
Female	52	42.6
Male	70	57.4
**Race**		
Asian	3	2.5
Black	4	3.3
More than one race	2	1.6
Not reported	1	0.8
White	112	91.8
**Dyad role**		
Care recipient	47	38.5
Care partner	75	61.5
**Care partner relationship**		
Spouse or partner	58	77.3
Child	12	16.0
Sibling	2	2.7
Other relative	2	2.7
Friend or neighbor	1	1.3
**Self-reported diagnosis**		
Dementia or Alzheimer disease	12	36.4
General memory problems	9	27.3
No dementia, Alzheimer disease, or general memory problems	12	36.4

### Measures

#### Purpose in Life

Purpose in life was measured by seven items from the Ryff Scales of Psychological Well-Being (1989). Participants rated their agreement with statements such as, “I live life one day at a time and do not really think about the future” or “They live life one day at a time and do not really think about the future” when responding to statements about the other member of the dyad. Responses were recorded using a seven-point Likert scale (1, *Strongly Disagree* to 7, *Strongly Agree*). Higher scores indicate a sense of purpose and more aims and objectives for living. Previous work with this scale has demonstrated its positive correlations with conceptually related well-being constructs (Ryff, 1989; Ryff and Keyes, 1995), as well as its associations with healthy aging outcomes (Windsor et al., 2015; Lewis et al., 2017). Reliability in the current sample was strong (*α* = 0.75; *α*_care-recipeints_ = 0.76; *α*_care-partners_ = 0.74).

##### Purpose Change Scores

Changes in ratings of purpose in life across the two timepoints were calculated by subtracting post-appointment scores from pre-appointment scores. A positive “change in purpose” score indicates that the person has declined in sense of purpose across the duration of the study.

##### Purpose Discordance Scores

Within dyads, other-ratings of purpose were subtracted from self-ratings of purpose in order to produce a “discordance score.” Discordance scores close to zero indicate relative similarity between the self- and other-rating; positive discordance scores indicate that the person reported a higher self-rating of purpose than the other-rating produced by their partner and negative discordance scores indicate that the person reported a lower self-rating of purpose than the other-rating produced by their partner. When appropriate, self- and other-ratings will take the form of “person being rated – person who is rating” (e.g., “care partner-other” refers to a rating of the care partner given by the other member of the dyad).

#### Diagnostic Experience

In the post-appointment interview, care recipients and care partners answered whether they thought the care recipient had memory issues and whether the issues would grow worse. Participants then answered either *yes*, *no*, or *I do not know* to indicate whether the doctor told them they had memory and thinking problems, dementia, Alzheimer disease, or another disorder. Based on their response to these questions, dyads were classified into three groups: those who were told they had Alzheimer disease or dementia, those who were told they had memory and thinking problems but were *not* told they had Alzheimer disease or dementia, and those who were told they had no objective issues with their memory or thinking.

### Procedure

Before their evaluation, care recipients and care partners were separately contacted to participate in the pre-appointment interview. The 15-min pre-appointment interview was conducted over the telephone before the scheduled appointment. Interviews took place between two and a half weeks before the appointment to earlier in the day on the day of the appointment. Dyads then attended the clinic for their memory evaluation, which included a review of medical history as well as a physical and neurological examination. At the conclusion of the evaluation, dyads were brought together for a feedback session during which the provider disclosed their diagnosis and prognosis. Following the evaluation, care recipients and care partners were called by telephone and separately completed the post-appointment interview. The 40-min post-appointment interview was conducted between 2 days and 2 weeks after the care recipient’s appointment. Individuals who completed the initial pre-appointment interview received a $5 gift card. Individuals who completed the second post-appointment interview received a $30 gift card, due to the longer length.

### Data Analysis

Analyses were conducted with R and RStudio. Descriptive statistics were calculated for demographic characteristics and dyad relationships. Paired-samples *t* tests were conducted to compare pre- and post-appointment responses and investigate change in purpose across the duration of the study while independent-samples *t* tests were used to compare self- and other-ratings between dyads. A one-way ANOVA was conducted to investigate potential differences in reported purpose change scores between diagnostic groups.

## Results

**Figure 1:**
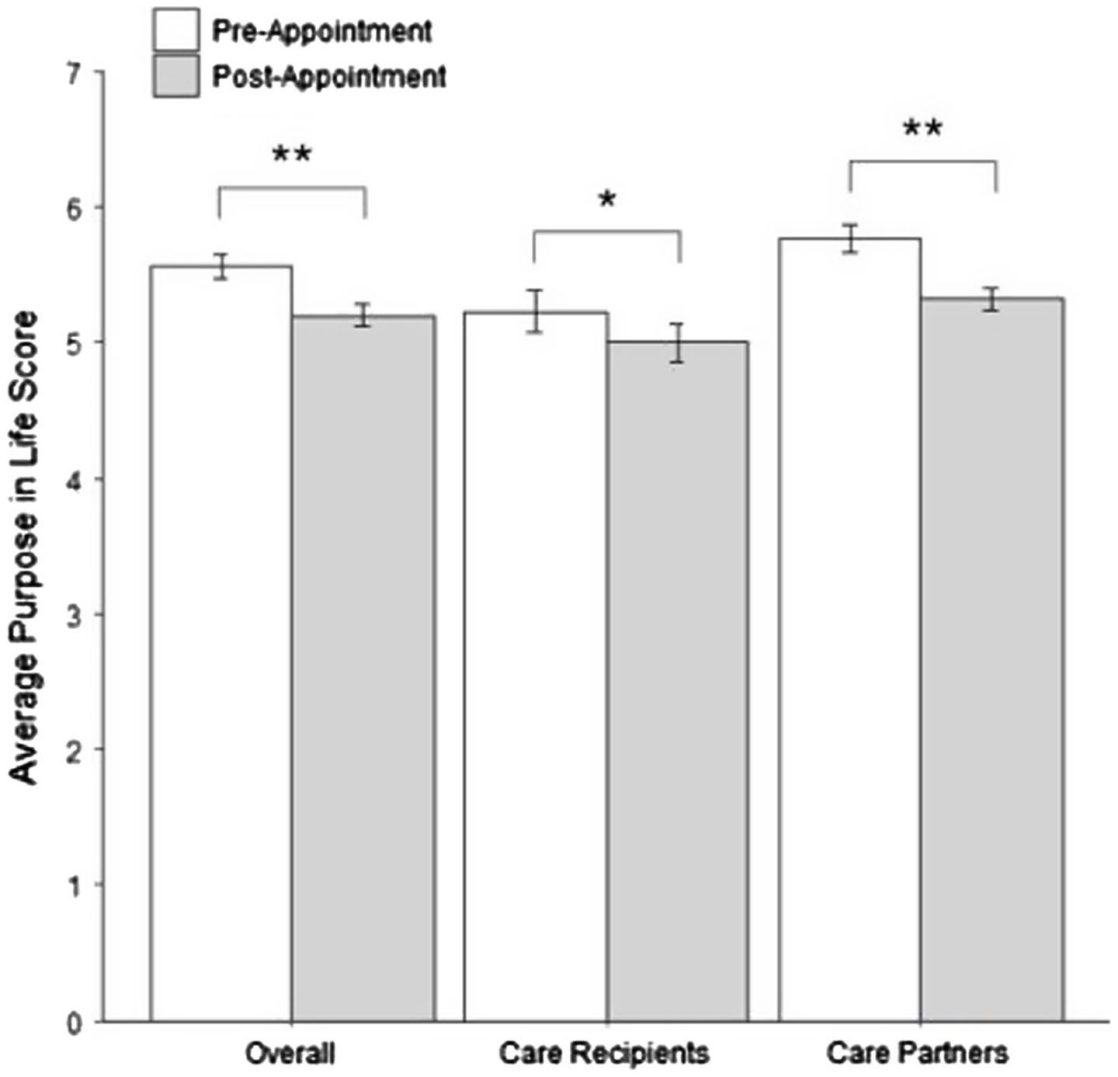
Change in self-reported purpose for care recipients and care partners. ^*^p < 0.01; ^**^p < 0.001.

Overall, self-reported purpose significantly decreased [*t*(85) = 7.01, *p* < 0.001] between pre- and post-appointment measurements (Figure 1). Participants, in general, declined approximately one-half of a point (*M*_change_ = 0.46 points) over the course of the study. This general trend held when analyses focused on only care recipients [*M*_change_ = 0.30 points, *t*(32) = 2.81, *p* = 0.008] and care partners [*M*_change_ = 0.56 points, *t*(52) = 6.93, *p* < 0.001]. Further analysis based on self-reported diagnosis received during the appointment revealed no significant differences in purpose change score [*F*(2, 72) = 0.029, *p* = 0.972]. Care recipients and partners appeared to report similar declines in purpose regardless of whether they reported being told they had Alzheimer disease or dementia (*M*_change_ = 0.46 points); memory and thinking problems (*M*_change_ = 0.44 points); or that they did not have any issues with dementia, Alzheimer disease, or any general memory problems (*M*_change_ = 0.48 points).

When comparing self- and other-ratings for each dyad, there were moderate positive correlations between self- and other-ratings of purpose using both pre-appointment (*r* = 0.49, *p* < 0.001) and post-appointment (*r* = 0.42, *p* < 0.001) measures. Overall, other-reported purpose did not show a significant change across the two timepoints [*M*_change_ = 0.09 points, *t*(84) = 1.03, *p* = 0.304]. Similarly, this lack of difference held when analyses focused on specific members of the dyad as both care partner purpose, rated by care recipient [*M*_change_ = −0.18 points, *t*(31) = −1.97, *p* = 0.057] and care recipient purpose, rated by care partners [*M*_change_ = 0.25 points, *t*(52) = 2.05, *p* = 0.045], showed small differences between pre-appointment and post-appointment ratings.

**Figure 2:**
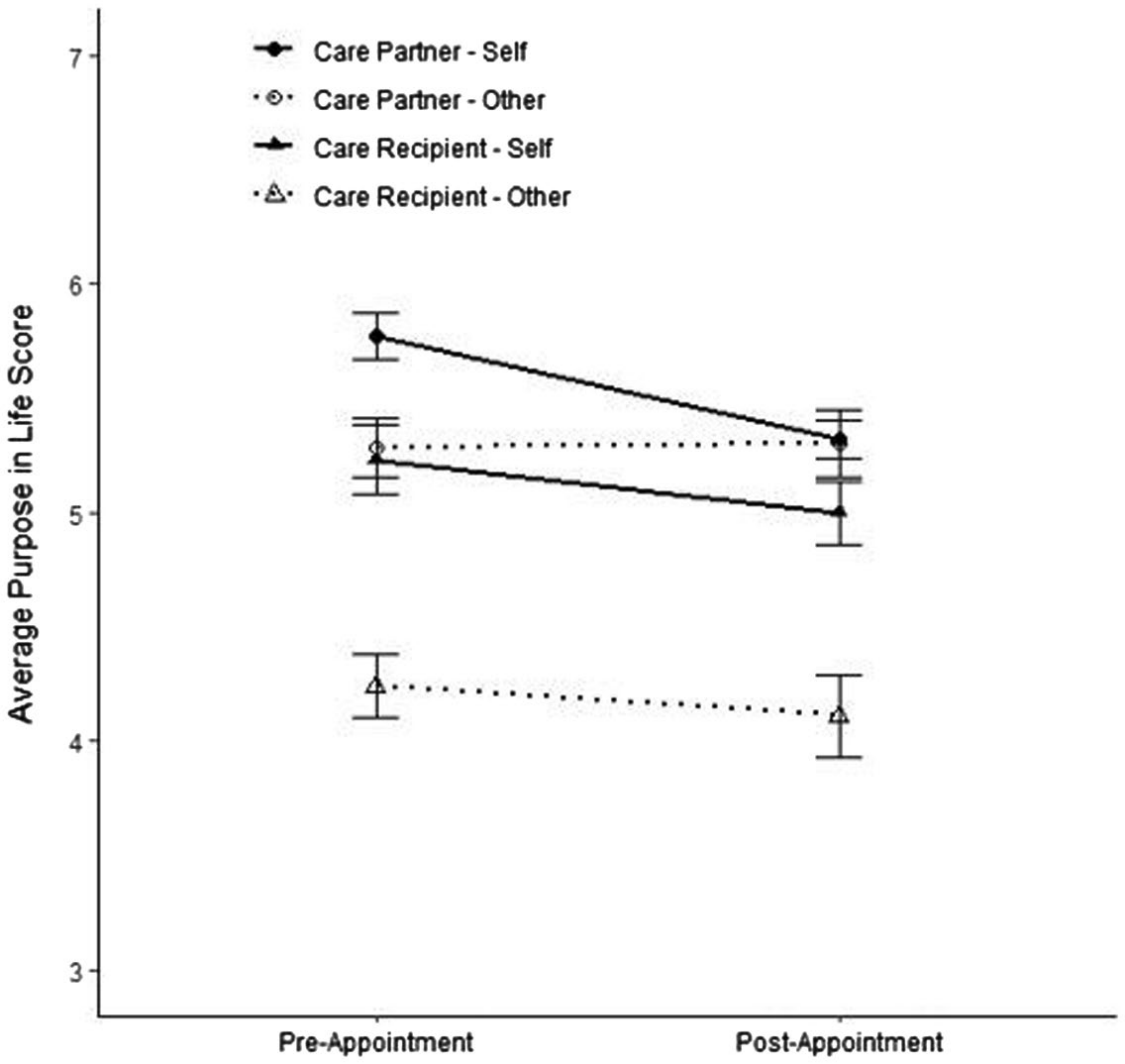
Comparisons of self- and other-reports of purpose for care partners and care recipients.

Finally, we explored whether care partners or care recipients displayed biased or inaccurate views of their partner’s sense of purpose. Discordance scores were calculated and support the idea that care partners underestimate the sense of purpose of their care recipients at both pre-appointment [*M*_discordance_ = 0.76, *SD*_discordance_ = 1.24, *t*(110.02) = −4.72, *p* < 0.001] and post-appointment [*M*_discordance_ = 0.54, *SD*_discordance_ = 1.27, *t*(86.98) = −3.96, *p* < 0.001] timepoints (Figure 2). On average, care recipients’ self-ratings of purpose were one-half to three-quarters of a point higher than the care partners’ other-ratings. Counter to expectations, analyses focusing on care partners’ purpose found similar differences prior to the appointment, insofar that recipients also seemed to underestimate levels [*M*_discordance_ = 0.52, *SD*_discordance_ = 0.90, *t*(91.77) = 3.02, *p* = 0.003]; however, self- and observer-ratings were relatively concordant [*M*_discordance_ = −0.07, *SD*_discordance_ = 0.83, *t*(51.90) = 0.14, *p* = 0.89] following the appointment (Figure 2).

## Discussion

The current study sought to explore changes in sense of purpose in both care recipients and care partners following a dementia diagnostic evaluation. Overall, we found that older adults who engaged in a diagnostic evaluation at a specialized memory clinic declined in their sense of purpose in life following their diagnostic appointment. A previous study (Hill et al., 2021) found that onset of illness did not change sense of purpose over 4 years, and previous work with care partners found that engagement in the care partner role may increase sense of purpose over time (Lang and Fowers, 2019). However, our findings may highlight negative self-reflection immediately following the appointment as both members of the dyad wonder how they are going to cope with their potentially different life ahead. Care recipients may wonder how their disease will progress and experience feeling like a burden to the care partner. Likewise, care partners may feel increased stress as they face the transition, often from spouse to caregiver, and wonder how their previously established life plans (e.g., retirement and travel) will be affected. Perhaps unexpectedly, a decline in self-rated purpose was observed for both care recipients and care partners, and it differed little depending on the diagnosis of dementia. This potentially points to the uncertainty that follows dementia diagnostic appointments, regardless of outcome. For those who receive a diagnosis, there is uncertainty regarding how the disease will progress and how their care partner will arrange care for the recipient. For those who do not receive a diagnosis, uncertainty remains over the cause of the symptoms that spurred their trip to the clinic and whether or not they will continue to seek assessment or treatment.

While care recipients and care partners self-report a decline in sense of purpose, they do not report a similar expected decline when rating the other member of the dyad. Care recipients’ other-reports were moderately correlated with self-report for the other member of the dyad, indicating that partners were moderately good judges of each other’s sense of purpose. However, our results indicate that care partners tend to underestimate the sense of purpose felt by the other member of their dyad. Care recipients, though they report similar declines in purpose to care partners, remain purposeful on average despite their role as the patient. This finding may be unexpected and somewhat surprising, considering the cognitive limitations faced by care recipients. However, our findings suggest that people with dementia still report better well-being than negative stereotypes may suggest, and better well-being than their care partners would estimate. Thus, our findings do not support the claim that individuals may feel purposeless in the face of subjective or objective cognitive decline, and the associated bias from others that may accompany a dementia diagnosis.

### Limitations and Future Directions

When interpreting the results, some limitations should be considered. Foremost, details of the care recipient’s diagnosis were not available from the memory clinic. Thus, we relied on the care recipient and care partner to communicate the diagnosis. Previous studies show that both care partners and recipients are relatively good reporters, especially for cases where diagnosis of no dementia or mild dementia are given (Zaleta et al., 2012). Although it is possible that participants misremembered the diagnosis the care recipient received, it seems more likely that what the care recipient or care partner perceived to be the true diagnosis – regardless of accuracy – would have the most significant influence on reported purpose. Additionally, due to a small sample size and a lack of power, we collapsed across diagnoses labels in a way that grouped Alzheimer disease and dementia diagnoses together. While Alzheimer disease is captured by the term dementia, there is nuance between these two diagnoses and future work should aim to distinguish between the labels.

Another limitation to consider is that the national prevalence of dementia varies by race and ethnicity (Plassman et al., 2007; Mehta and Yeo, 2017), which we were unable to account for given our sample was disproportionately White. Therefore, our results may not be generalizable to the diverse population of Americans with dementia. Furthermore, the participants in the present study were recruited from a tertiary memory clinic. Tertiary care providers tend to serve broader geographic areas due to the specialized nature of their care, and long travel times are often required of the care recipient and care partner (Newington and Metcalfe, 2014). Consequently, those who seek tertiary health care services may have more time or financial resources than the general population of individuals with dementia.

Finally, we collected reports of purpose just prior and just following the diagnostic appointment. Caregiving for a person with dementia is itself an other-focused act but some researchers cite a sense of meaning and purpose for the caregiver as a potential motivating factor in providing care to a person with dementia (Hill et al., 2020). It is possible that the initial downward trajectory in purpose demonstrated in this study may change as care partners find purpose in the caregiving process over time. Obtaining reports further out from the appointment would be a valuable addition to any future studies.

In conclusion, care recipients and partners were generally purposeful, but both reported less purpose in life following their dementia diagnostic appointment. When asked to report on the other member of the dyad, care partners tended to be biased toward care recipients and assumed their purpose would be significantly lower than care recipients reported, illustrating that older adults with cognitive concerns are more purposeful than expected. Additional studies with larger, more diverse samples in term of both demographics and diagnosis are warranted to more fully examine the effect of a dementia diagnostic appointment on a person’s sense of purpose in life.

## Data Availability Statement

The raw data supporting the conclusions of this article will be made available by the authors, without undue reservation.

## Ethics Statement

The studies involving human participants were reviewed and approved by Human Research Protection Office Washington University in St. Louis. The patients/participants provided their written informed consent to participate in this study.

## Author Contributions

MW led data collection, data analysis, and writing of the manuscript. MW and PH conceived of the presented idea, designed, and directed the project. CJ contributed to the collection of the data, analysis of results, and to the writing of the manuscript. All authors contributed to the article and approved the submitted version.

## Conflict of Interest

The authors declare that the research was conducted in the absence of any commercial or financial relationships that could be construed as a potential conflict of interest.

## Publisher’s Note

All claims expressed in this article are solely those of the authors and do not necessarily represent those of their affiliated organizations, or those of the publisher, the editors and the reviewers. Any product that may be evaluated in this article, or claim that may be made by its manufacturer, is not guaranteed or endorsed by the publisher.
